# Neuropsychological Functioning in Obsessive-Compulsive Washers: Drug-Naive Without Depressive Symptoms

**DOI:** 10.18869/nirp.bcn.8.3.233

**Published:** 2017

**Authors:** Ali Akbar Saremi, Seyed Vahid Shariat, Mohammad Ali Nazari, Behrooz Dolatshahi

**Affiliations:** 1. Department of Cognitive Neuroscience, Institute for Cognitive Science Studies (ICSS), Tehran, Iran.; 2. Mental Health Research Center, Tehran Institute of Psychiatry, School of Behavioural Sciences and Mental Health, Iran University of Medical Sciences, Tehran, Iran.; 3. Department of Psychology, Faculty of Education & Psychology, University of Tabriz, Tabriz, Iran.; 4. Department of Psychology, University of Social Welfare and Rehabilitation Sciences, Tehran, Iran.

**Keywords:** Obsessive-Compulsive Disorder, Washers, Neuropsychology, State-trait deficits

## Abstract

**Introduction:**

Obsessive–Compulsive Disorder (OCD) is a complex and heterogeneous neuropsychiatric syndrome. Contamination obsessions and washing/cleaning compulsions are the most frequent clinical OCD subtypes. The current study aimed at examining the neuropsychological impairments in drug-naive obsessive-compulsive (OC) washers without depressive symptoms and their association with the severity of symptoms.

**Methods:**

In the current causal-comparative study, 35 patients with diagnostic and statistical mental disorders class (DSM)-IV diagnosed with washing-subtype OCD and 35 healthy subjects were selected by the convenience sampling method and evaluated by computerized neuropsychology battery and clinical tests as Stroop Color-Word Test (SCWT), Wisconsin Card Sorting Test (WCST), Go/No-Go Test, Digits Forward (DF), Digits Backward (DB), Yale-Brown Obsessive–Compulsive Scale (Y-BOCS), Beck Depression Inventory (BDI), Beck Anxiety Inventory (BAI), and General Health Questionnaire (GHQ)-28. The patients were matched to the comparison group with regard to age, gender, intelligence quotient (IQ), education, and handedness. All the tests were standardized in Iran. SPSS version 20.00 was used for descriptive and analytical data analysis.

**Results:**

There was no statistically significant different between the OCD washing and the control groups regarding socio-demographic variables or IQ. There were significant differences between the OC washer and the healthy control groups on the neuropsychological functioning. The obtained results suggested that OC washers performed significantly worse on neuropsychological measures than the controls. There was no significant association between the severity of OC symptoms and the neuropsychological functions in the OCD washing group.

**Conclusion:**

It was concluded that executive function impairment, which is a core feature in OC washers was trait-like in nature.

## 1. Introduction

OCD is an incongruous and heterogeneous disorder with different subtypes (Mataix-Cols, Pertusa & Leckman, 2007). The most common subtypes consist of 1) Symmetry obsessions and repeating, ordering, and counting compulsions; 2) Forbidden thoughts (aggression, sexual, religious, or somatic) and checking compulsions; 3) Contamination/washing, and 4) Hoarding obsessions and collecting compulsions (Bloch, Landeros-Weisenberger, Rosario, Pittenger, & Leckman, 2008; Mataix-Cols, Nakatani, Micali, & Heyman, 2008). Notably, some recent researches suggested responsibility for harm instead of hoarding (Abramowitz et al., 2010). Contamination obsessions and washing/cleaning compulsions are the most frequent clinical subtypes of OCD with 45% to 60% prevalence (Matsunaga, Hayashida, Kiriike, Maebayashi, & Stein, 2010; Wang et al., 2012; American Psychiatric Association, 2013). The main characteristics of the OC washers are intrusive thoughts about contamination, ritualized washing/cleaning behaviors, and emotions of fear and disgust (Leopold & Backenstrass, 2014).

In the past 2 decades, the researchers reported that the neuropsychological deficit was one of the main characteristics of patients with OCD (Shin, Lee, Kim, & Kwon, 2014; Abramovitch, Abramowitz, & Mittelman, 2013; Snyder, Kaiser, Warren, & Heller, 2015). The neuropsychological studies show that patients with OCD have deficits in motor inhibition (Chamberlain et al., 2007; Chamberlain, Blackwell, Fineberg, Robbins, & Sahakian, 2006; Bannon, Gonsalvez, Croft, & Boyce, 2006; Penades et al., 2007; Abramovitch, Dar, Schweiger, & Hermesh, 2011; da Rocha, Alvarenga, Malloy-Diniz, & Correa, 2011; Ghisi, Bottesi, Sica, Sanavio, Freeston, 2013), cognitive inhibition (Penades et al., 2007; Bannon et al., 2006), reaction time (Rauch et al., 2007), verbal memory (Moritz, Kloss, von Eckstaedt, & Jelinek, 2009), nonverbal memory (Simpson, et al., 2006), problem solving (Cavedini, Zorzi, Piccinni, Cavallini, & Bellodi, 2010; Starcke, Tuschen-Caffier, Markowitsch, & Brand, 2010), and set-shifting (Ghassemzadeh et al., 2007; Nejati, Zabihzadeh, Maleki, & Safarzadeh, 2013; Hekmati, Hashemi, & Pirzadeh, 2012; Bannon et al., 2006; Tukel et al., 2012; Aigner et al., 2007; Bucci et al., 2007). These deficits can have a determining role in incidence, maintaining, and severity of OCD clinical symptoms.

Neurocognitive executive functions are the high level functions of neural system referred to a set of cognitive abilities including planning, set-shifting and problem solving ability, cognitive flexibility, and rule acquisition (Rieger, 2000; Nakao, Okada, & Kanba, 2014).

In comparative studies between normal individuals and patients with OCD, it was found that patients with OCD had deficits in several executive functions such as set-shifting, working memory, conflict resolution/response inhibition, and decision making (Penades, Catalan, Andres, Salamero, & balGasto, 2005; Nejati et al., 2013; Kashyap, Kumar, Kandavel, & Reddy, 2013; Tukel et al., 2012). Cognitive flexibility, which is mediated by frontostriatal loops (Kehagia, Murray & Robbins, 2010), is the ability to adapt to the environment changes. Set-shifting is defined by Abramovich and Cooperman (2015), as “to continuously disengage from irrelevant stimuli or information while engaging in relevant task features”. Patients with OCD show deficient set-shifting on the Wisconsin Card Sorting Test (WCST) (Okasha et al., 2000; Aigner et al., 2007; Bucci et al., 2007; Ghassemzadeh et al., 2007; Hekmati et al., 2012; Tukel et al., 2012).

Inhibitory deficit in patients with OCD may be considered as the core clinical symptoms of OCD (Chamberlain, Blackwell, Fineberg, Robbins, & Sahakian, 2005). It includes 2 types of inhibitions, cognitive and behavioral (Shaffer & Kipp, 2007). The cognitive inhibition is the process of preventing unrelated information to enter into the working memory (Nigg, 2000); while the behavioral inhibition is the individual’s ability to prevent an activity, and stop or delay an action (Clark, 1996). Omission errors (failing to respond to a go stimuli) on the Go/No-Go Test is usually used to assess sustained attention.

Commission error (responding to no-go stimuli) on Go/No-Go Task is considered as a gold standard indicator of response inhibition (Abramovitch & Cooperman, 2015). Decreased activation of the Dorsolateral Prefrontal Cortex (DLPFC), Inferior Frontal Gyrus (IFG), striatum, and thalamus in patients with OCD during inhibition is reported by several neuroimaging studies (Roth et al., 2007; Woolley et al., 2008; Page et al., 2009; Rubia et al., 2009; De Wit et al., 2012). Working memory identified as the ability to store and manipulate information for a short time is in association with DLPFC and other prefrontal regions (Nee et al., 2013; Abramovitch & Cooperman, 2015).

Neuropsychological deficits may play a mediating role between brain dysfunctions and clinical symptomatology (Savage, 1998; Olley, Malhi, & Sachdev, 2007; Chamberlain et al., 2008; Harrison et al., 2009). Results of several studies indicated no significant correlation between the severity of OC symptoms and performance in neuropsychological test (Bedard, Joyal, Godbout, & Chantal, 2009; Bucci et al., 2007); whereas some other researchers reported a significant correlation between them (Abramovitch et al., 2011; Nedeljkovic et al., 2009; Segalas et al., 2008). Despite the existence of neuropsychological evidence on the weak executive function in patients with OCD, no systematic study is conducted on the nature of neuropsychological deficits in OCD washing. Such studies can determine the symptomatological dependence of these deficits and classify them into trait-related (independent) and state-related (dependent) subtypes. Moreover, determination of the nature of the neuropsychological deficits, in addition to help better decision-making in clinical situations, could pave the way to understand the psychopathological basis of this disorder, which would be useful in prevention and treatment programs of OCD washing.

The inconsistencies across neuropsychological studies on the patients with OCD necessitate the investigation on potential confounding factors of neuropsychological function. Understanding the relationship between OCD and depression may be useful in terms of symptomatology, etiology, neuropsychology, neuroimaging, and in particular treatment of the comorbidity of anxiety (63.3%), mood (75.8%), and disorders in OCD (Ruscio, Stein, Chiu & Kessler, 2010; Klein Hofmeijer-Sevink et al. 2013). Some studies suggested that the neuropsychological differences between the patients with OCD and healthy subjects were because of co-occurrence of depression in such patients (Basso, Bornstein, Carona, & Morton, 2001; Moritz et al., 2001; Moritz et al., 2005).

While several researches did not report depression or the severity of its symptoms as the moderating factor in OCD (Nedeljkovic et al., 2009; Abramovitch et al., 2011; Hekmati et al,. 2012; Penades et al., 2005), some studies suggested that the neuropsychological differences between the patients with OCD and healthy subjects can be due to the potential effect of medication. In this regard, some studies reported neuropsychological functions deficits in the patients with OCD with medication (Basso et al., 2001; Moritz et al., 2002; Kuelz, Hohagen, & Vodereholzer, 2004), and other studies did not report any deficits in the patients with OCD without medication (Sieg, Leplow, & Hand, 1999; Krishna et al., 2011). Hence, the mentioned study controlled the depression variable and medication effect on the comorbidity of anxiety and depression in patients with Parkinson disease.

OCD washing subtype is distinguishable from other OCD subtypes from neuropsychological, neurobiological, behavioral, and cognitive aspects (Broderick, Grisham, & Weidemann, 2013). To the best of the authors’ knowledge, so far no research in Iran was conducted on the drug-naive OC washers without depression symptoms regarding the neuropsychological dysfunctions. Such a study on Iranian patients with OCD is of particular importance as OCD washing is one of the most common ritual behaviors in Iranian population (Ghassemzadeh, Khamseh, & Ebrahimkhani, 2005) due to religious and cultural reasons.

The neuropsychological researches mostly used the analogue OCD samples or mixed clinical samples of various subtypes of the patients with OCD; this kind of sampling limits the generalizability. Therefore, the current study particularly investigated the neuropsychological functions of the OC washers by controlling the possible confounding effects of depression and medication. To obtain more precise information, reduce anxiety and the interaction between the examinee and examiner, a computerized neuropsychological battery was applied. The current study particularly investigated the neuropsychological performances mostly independent of verbal functioning. Identifying the neuropsychological functions of the OC washers can lead to reconsideration in the existing theories and viewpoints of OCD washing.

The current study mainly aimed at comparing the neuropsychological functions between the OC washers who were drug-naive without depression symptoms and the healthy controls, and also assessing the correlation between OC symptoms and neuropsychological functions. It was hypothesized that patients with OCD washing perform weaker than the healthy controls on tests of set-shifting, response inhibition (cognitive and behavior), attention, and working memory.

## 2. Methods

### 2.1. Participants

A total of 35 patients with OCD (pure washers) (26 females and 9 males) and the same number of healthy subjects were recruited to for the present study. The 2 groups were matched in age (ranged from 20 to 40 years old), gender, Intelligence Quotient (IQ), education, and handedness. Patients with OCD were selected from Pardis Private Psychotherapy Centre in Mashhad, Iran, from 2013 to 2015. Patients with OCD washing received a standardized clinical interview (the semi-structural format for diagnostic and statistical manual of mental disorders (DSM)-IV, 2000 Persian edition) (Mohamadkhani, Jookar, Jahantabesh, & Tamanaeiifar, 2010). The patients were excluded from the study if they had co-occurred psychiatric disorders, substance abuse, personality disorder, as well as head injury, medical and neurological diseases, and low IQ (<90). The Yale-Brown obsessive–compulsive scale (Y-BOCS), Beck Anxiety Inventory (BAI), and Beck depression inventory (BDI)-II were also administered for the participants with OCD. Patients with a total Y-BOCS≥16 were included. The case and control groups were selected by the convenience sampling method and accurate matching process. Control group was selected by the research assistants from family and friends. No patients’ family members were recruited for the control group. Normal group with a total General Health Questionnaire (GHQ) score≥24 were excluded. The total GHQ score of 24 was considered as cutoff point to classify significant social distress. For all subjects, the intelligence test was performed; right handedness was confirmed and the minimum education level was kept at the 9^th^ grade.

### 2.2. Measures

#### 2.2.1. Demographics and clinical information questionnaire

Demographical and clinical data (gender, age, educational status, duration of disorder, onset of the symptoms, history of treatment, kind of obsessions and compulsions) and IQ score were recorded. The Standard Progressive Matrices (SPM) (Raven, 1991) test was used to measure the intelligence.

#### 2.2.2. Edinburgh Handedness Inventory

The validity and reliability of EHI (Oldfield, 1971) are confirmed by many studies (Dorthe, Blumenthal, Jason, & Lantz, 1995; Ransil & Schachter, 1994). Test-retest reliability of the EHI is reported ranging from 0.95 to 0.98 (Ransil, & Schachter, 1994). All subjects were checked by the Persian version of EHI.

#### 2.2.3. Yale-Brown Obsessive-Compulsive Scale

This scale is widely known as gold standard to evaluate the symptoms improvement in patients with OCD (Steketee, Frost, & Bogart, 1996). The Y-BOCS (Goodman et al., 1989) includes 10 self-report items to independently assess 2 aspects of OCD: obsession (items 1 to 5) and compulsion (items 6 to 10). The scores of the items were measured based on a 5-point Likert scale from 0 (no symptoms) to 4 (extreme symptoms). The Y-BOCS showed excellent internal consistency (Cronbach’s alpha=0.88 to 0.91) and good validity (Goodman et al., 1989). The Persian version (Rajezi Esfahani, Motaghipour, Kamkari, Zahiredin, & Janbozorgi, 2012) suggests the cutoff point of 9 to distinguish the patients from healthy people with excellent internal consistency (Cronbach’s alpha=0.97). Its score ranged from 0 to 40. Classification of scores was as follows: subclinical (0 to 7), mild (8 to 15), moderate (16 to 23), severe (24 to 33), and extreme (34 to 40).

#### 2.2.4. Beck Depression Inventory (2^nd^ edition)

The BDI-II (Beck, Steer, & Brown, 1996; BDI-II) was used to determine the severity of depressive symptoms. The Persian version (Ghassemzadeh, Mojtabai, Karamghadiri, & Ebrahimkhani, 2005) had excellent internal consistency (Cronbach’s alpha=0.87). The validity and reliability of BDI-II were reported as 0.84 and 0.70, respectively, in Iran (Goudarzi, 2001). Classification of scores was as follows: minimal (0 to 13), mild (14 to 19), moderate (20 to 28), and severe (>29).

#### 2.2.5. Beck Anxiety Inventory

The BAI (Beck & Steer, 1993) was used to assess severity of anxiety symptoms. The Persian version of BAI (Kaviani & Mousavi, 2008) proved a good reliability (r=0.72), a very good validity (r=0.83), and an excellent internal consistency (Cronbach’s alpha=0.92). Classification of scores was as follows: minimal (0 to 7), mild (8 to 15), moderate (16 to 25), and severe (26+).

#### 2.2.6. General Health Questionnaire

The GHQ-28 (Goldberg and Hillier, 1979) was used to investigate the health status of the normal group. The highest scores indicated poor psychological well-being of the subject. Each item was scored from 0 to 3, resulting in total possible score range of 0 to 84; the total score of 24 was considered as cutoff point to classify “significant social distress” (Sterling, 2011). The reliability of the Persian version was evaluated by Taghavi (2001) applying test-retest (0.70), split-half (0.93), and Cronbach’s alpha (0.90) tests.

#### 2.2.7. Stroop Color-Word Test

The SCWT (Lezak, Howieson, & Loring, 2004; Stroop, 1935) was developed to assess cognitive flexibility, selective attention, and inhibitory control (Lezak et al., 2004). This test is a good measure of cognitive inhibition. The Persian version of SCWT adapted to Iranian population was employed in the current study. Ghadiri, Jazayeri, Ashayeri, and Ghazi Tabatabaei (2006) reported that the internal consistency of reaction times were 0.6, 0.83, and 0.97 in each stage, respectively. Internal consistency for errors numbers were also 0.55, 0.78, and 0.79 in each stage respectively. The current study used the software developed by Ravan Tajhiz Sina Company. It takes 2 seconds to present each stimulus on the screen, with the presentation interval of 800 ms between the 2 stimuli.

The stimuli are words with 2 dimensions (the form of the word and the color of the ink). The answers could be congruent (congruency between the meaning of the word and the color the ink), or incongruent, (differencing between the meaning of the word and the color the ink). Stroop interference effect is calculated as the sum of the 2 indices: Stroop effect and Stroop error. Stroop effect is defined as the difference between the mean reaction time to incongruent and congruent trials; while Stroop error is calculated as the difference in the mean number of incongruent and congruent responses.

#### 2.2.8. Wisconsin Card Sorting Test

The WCST was used to determine abstract behavior, set-shifting, mental flexibility, as well as sustained attention (Lezak et al., 2004). A short-form (64 image) of the WCST (Kong, Thompson, Iverson, & Heaton, 2000) contains images in different shapes (cross, circle, triangle, and/or star-shaped), numbers (1 to 4), and colors (red, yellow, blue, and/or green). The current study used the WCST software version 64 developed by Ravan Tajhiz Sina Company. The interval between the end of feedback and the next card appearance was 700 ms, feedback length was 200 ms, and the interval between the subject’s response and the feedback was 100 ms. Naderi (1994) obtained test-retest reliability of 0.85 among Iranian population. The categories, total, and preservative errors were the outcome measures used in the current study.

#### 2.2.9. Digit Span Test

The test (Wechsler, 1997) has 2 parts: digit forwards (DF) and digit backwards (DB); the first part has 8 2-trial items, while the latter includes 7 ones. As their titles reveal, DF includes forward-ordered sequence of numbers, but reverse-ordered in DB. Each correct sequence corresponds to a point giving rise to a 0 to 15 score range. The DF is considered as a measure of global attention, whereas the DB is associated with working memory (Tukel et al., 2012).

#### 2.2.10. Go/No-Go Task

Go/No-Go Task is a commonly used test of behavioral inhibition (Bannon, Gonsalvez, Croft, & Boyce, 2002). Ghadiri et al. (2006) reported test-retest reliability of 0.87 in a sample of Iranian population. The current study used the computerized version of the program (Super lab Pro 4.0). It takes 2 seconds to present each stimulus on the screen, with the presentation interval of 500 ms between 2 stimuli. In the current study, 100 “go” stimuli and 100 “no-go” were shown randomly. Red and green circles were presented to the subjects. During each condition, the green/red stimuli were randomly shown. The results expressed as omission and commission errors for “go” and “no-go” stimuli, respectively. Three performance indices were scored: reaction time, omission and commission errors.

### 2.3. Procedure

After a general explanation about the research purpose, participants signed the consent form. All participants completed a demographic questionnaire. SPM (Raven, 1991) and EHI were performed for all participants. The subjects with OCD were interviewed and afterwards BAI, Y-BOCS, and BDI-II were completed. GHQ was administered among the normal participants. Finally, the computerized neuropsychological battery was conducted. Single-session neuropsychological assessment (for 2 or 3 hours) was done on all subjects in a fixed sequence and a same quiet environment. If necessary, the subjects were allowed to rest in a short break in the middle of measurements. It should be noted that all percipients voluntarily contributed to the study and ethical considerations of the research were investigated and approved by the Review Board of the Institute for Cognitive Science Studies, Tehran, Iran. All the information was kept confidential. Patients with the OCD were then referred to the psychiatry for pharmacological treatment.

### 2.4. Data analysis

The statistical analysis was carried out by Statistical Package for the Social Sciences (SPSS) version 20 (Chicago, IL) software. The normality of data was confirmed by the Kolmogorov–Smirnov test. Group differences regarding demographics (age and education), and IQ were analyzed using independent samples t test. To examine the differences between the groups on neuropsychological test variables, multivariate analysis of variance (MANOVA) was conducted. MANOVA is suitable to make a comparison between the groups in terms of various features, particularly if there are several dependent variables. The homogeneity of variance and covariance pre-assumptions were tested using the Levene and Box’s M tests, and the assumptions were met. Then, the data were analysed. Univariate analyses were performed for each of the neuropsychological test variables. Type I errors with the Bonferroni correction were controlled. Effect sizes (Cohen’s d) were additionally calculated for all neuropsychological parameters. Further, the Pearson correlation was used to examine the relationship between Y-BOCS scores and neuropsychological test variables in the washers group. Finally, partial correlation was applied to control the severity of depressive symptom.

## 3. Results

### 3.1. Sample characteristics

Descriptive analysis was conducted to examine demographic and diagnostic characteristics. Mean score and standard deviation (SD) of clinical and demographic features of OCD washers and control groups are shown in Table 1. There was no significant difference between the 2 groups in terms of age, gender ratio, educational status, and IQ. The mean Y-BOCS score was 25.03±1.82 (the severe degree of illness) indicating marked psychopathology. Mean age of OCD onset was 24.43±5.74 years and patients had OCD for almost 7.80±3.08 years on average. The mean total BDI-II score among the patients with OCD was 13.09±2.03. The BDI-II scores in the OCD group (Table 1) represent minimal severity. The mean total BAI score among the patients with OCD was 20.83±3.07. The BAI scores in the patients with OCD (Table 1) represent the moderate severity. Classification of scores was as follows: minimal (BDI 0 to 13, BAI 0 to 7), mild (BDI 14 to 19, BAI 8 to 15), moderate (BDI 20 to 28, BAI 16 to 25), and severe (BDI 29 to 63, BAI 26 to 63). In accordance with the findings of previous study (Labad et al., 2008) indicating dominance of females in OCD washing, the current study also showed higher number of female subjects with OCD.

**Table 1.** Demographic and clinical features of the study participants.

**Variable**	**OCD Patients (N=35)**		**The Controls (N=35)**		**Significance**

	**Mean**	**SD**	**Mean**	**SD**	**T (P)**
Age	32.45	4.74	32.61	5.00	−0.142(0.877)
Gender (% female)	74%	-	74%		
Education (years)	13.40	2.27	13.20	2.08	−0.383(0.703)
IQ (general)	109.54	4.55	110.03	4.85	−0.432(0.667)
Age at onset of symptoms	24.43	5.74			
Duration of symptoms (years)	7.80	3.08			
Y-BOCS total score	25.03	1.82			
Y-BOCS obsession	12.66	1.18			
Y-BOCS compulsion	12.37	1.30			
BDI-II total score	13.9	2.03			
BAI total score	20.83	3.07			

OCD: Obsessive-Compulsive Disorder; IQ: Intelligence Quotient; Y-BOCS: Yale-Brown Obsessive-Compulsive Scale; BDI-II: Beck Depression Inventory (second edition); BAI; Beck Anxiety Inventory.

### 3.2. Neuropsychological Functioning

The results of MANOVA test, based on the Wilk Lambda scale, showed a significant multivariate group effect [WCST (λ=0.225, P=0.001), Go/No-Go Task (λ=0.331, P=0.001), SCWT (λ=0.318, P=0.035), and digit span test (λ=0.341, P=0.001)]. The covariance matrices of the dependent variables were equal in both groups WCST (M=2.010, F=0.319, Sig.=0.927), Go/No-Go Task (M=10.632, F=1.687, Sig.=0.120), SCWT (M=3.620, F=1.168, Sig.=0.320), digit span test (M=1.345, F=0.434, Sig.=0.729) (the Box test). Type I errors with the Bonferroni correction were controlled. Thus, the Alpha levels of the tests were as follows: WCST P<0.016; Go/No-Go Task P<0.016; SCWT P<0.025; digit span test P<0.025. Significant differences in results of the univariate analysis for different measures could be interpreted as the sign of impartment of the neuropsychological Functions among OCD washers (Table 2).

**Table 2.** Differences between the study groups regarding neuropsychological variables.

		**The Case Group (N=35)**		**The Control Group (N=35)**		**F(1.68)**	**Significance**	**Effect Size Cohen’s d**

		**Mean**	**(SD)**	**Mean**	**(SD)**			
WCST	Perseveration error	11.03	2.04	6.69	2.30	70.02	0.001**	1.99
	Total error	19.77	2.87	14.26	2.88	64.28	0.001**	1.92
	Categories	3.49	0.85	5.14	0.88	64.04	0.001**	1.90
Go/No-Go Task	Omission errors	7.06	0.94	6.31	1.08	9.458	0.003*	0.74
	Commission errors	17.20	1.88	13.49	2.01	64.04	0.001**	1.90
	Reaction time	392.09	20.02	339.49	31.13	70.69	0.001**	2
SCWT	Stroop effect	119.63	10.98	96.54	12.14	69.62	0.001**	1.99
	Stroop errors	5.06	1.11	3.26	0.82	59.71	0.001**	1.84
Digit span test	Digit span forward	5.03	0.62	6.29	0.71	62.452	0.001**	1.89
	Digit span backward	4.25	0.61	5.37	0.68	51.202	0.001**	1.73

WCST: Wisconsin Card Sorting Test; SCWT: Stroop Color Word Test; Stroop effect, difference in mean reaction time between the incongruent and congruent responses (ms); Stroop errors: difference in mean number between the incongruent and congruent responses;

* P≤0.05,

** P≤0.01.

Set Shifting (WCST): Table 2 shows that the OCD washing and healthy groups were significantly different in the set shifting; therefore, they showed more perseverative errors, F(70.02), P=0.001, ES(1.99) total errors F(64.28), P=0.001, ES(1.92), and achieved fewer categories, F(64.04), and P=0.001, ES(1.90). OCD washing in the current study had a weaker performance in WSCT compared to the control group. They showed more preservative errors, total errors, and fewer completed categories.

Cognitive inhibition (Stroop task): As presented in Table 2, differences were also found between the case and control groups in terms of Stroop effect, F(69.62), P=0.001, ES(1.99), and Stroop errors F(59.71), and P=0.001, ES(1.84). OCD washing in the current study had a weaker performance in SCWT compared to that of the control group.

### 3.3. Behavior Inhibition (Go/No-Go Task)

As presented in Table 2, there were differences between the case and control group. For the no-go stimuli, the case group had a higher number of commission errors F(64.04), P=0.001, ES(1.90) and omission errors F(9.45), P=0.003, ES(0.74) than the control group. Also differences were observed between the OCD washers and the healthy control group in terms of reaction time F(70.69), P=0.001, ES(2). OCD washing in the current study had a weaker performance in go/no-go compared to the control group.

Global attention and working memory (digit span test): Table 2 shows that both groups significantly differed on digit span forward, F(62.45), P=0.001, ES(1.89) and digit span backward, F(51.20), P=0.001, ES(1.73). Large effect sizes (from 0.74 to 2) were observed in the differences between the case and the control groups (Table 2). According to Cohen (1988), effect size values of 0.2, 0.5, and 0.8 represent small, medium, and large effects, respectively. As shown in Table 3, there was no significant correlation between the severity of OC symptoms and neuropsychological functions (P>0.05). Partial correlation results showed no confounding effects of depression (P>0.05).

**Table 3.** Association of Y-BOCS score of the case group with the Neuropsychological Test variables and partial correlation coefficients of the BDI-II Scores.

	**Y-BOCS Total Score**	**Y-BOCS Obsessions**	**Y-BOCS Compulsions**
Perseveration error	−0.262(−0.232)	−0.130(−0.143)	−0.183(−0.178)
Total error	−0.156(−0.143)	−0.063(−0.21)	−0.274(−0.231)
Categories	−0.312(−0.288)	−0.227(−0.183)	0.229(−0.216)
Omission errors	−0.168(−0.161)	−0.124(.12478)	−0.018(0.919)
Commission errors	−0.124(−0.112)	−0.114(−0.112)	−0.111(.1125)
Reaction time	−0.241(−0.23)	−0.207(−0.233)	−0.130(−0.140)
Stroop effect	−0.174(−0.162 )	−0.270(−0.227)	−0.112(−0.12)
Stroop errors	−0.306(−0.247)	−0.141(−0.134)	−0.299(−0.281)
Digit span forward	−0.157(−0.146)	−0.147(−0.13)	−0.186(−0.162)
Digit span back ward	−0.192(−0.183)	−0.199(−0.18)	−0.186(−0.162)

Y-BOCS: Yale-Brown Obsessive Compulsive Scale; Partial correlations (for BDI-II scores) are given in parenthesis.

## 4. Discussion

The clinical and neuropsychological evidence showed that the patients with OCD had more neuropsychological deficits than normal individuals. The current study aimed at comparing the neuropsychological functions in the patients with drug naive OCD washing without depression symptoms with the healthy control group. The results showed that the patients with drug naive OCD washing without depressive symptoms had poorer performance than the control group in the cognitive and behavioral inhibition, the cognitive flexibility/set-shifting and abstractive ability. Moreover, depressive symptom severity was not associated with neuropsychology function.

Moritz et al. (2001) showed that the patients with OCD with depressive symptoms had cognitive deficits; while the patients with OCD with low depression scores were not significantly different from the healthy cases in executive functions. Some studies showed that the patients with OCD with depression symptoms did not report executive functions deficit (Nedeljkovic et al., 2009; Abramovitch et al. 2011). Hekmati et al. (2012) reported that the patients with OCD without depression symptoms had a weaker performance than the control ones in the executive functions such as inhibition, set shifting, and updating of working memory. Krishna et al. (2011), showed that there was no significant difference between the patients with OCD without depressive symptoms (n=30) and the healthy ones (n=30) in the majority of the executive functions. However, results of the study by Roopesh, Reddy, and Mukundan (2013) showed deficits in executive functions of the drug-naive patients with OCD without depressive symptoms.

Mortiz et al. (2005) indicated that neurocognitive deficits were associated with depressive symptoms. Kuelz et al. (2004) showed that the patients with OCD with medication had a weaker performance than the patients with OCD without medications, in neuropsychological functions. The inconsistent results reported by previous studies could be attributed to heterogeneity of OCD symptoms, matching criteria, comorbidities and more importantly medication history. Moreover, more data are needed to shed light on differences between OCD washing with or without depression.

OCD washing in the current study had a weaker performance in WSCT compared with the control ones. They showed more preservative errors, total errors, and fewer completed categories. The current study results indicated that the OCD washing had deficit in the cognitive flexibility ability/set-shifting and abstraction ability. The current study findings were consistent with many previous studies (Ghassemzadeh et al., 2007; Nejati et al., 2013; Hekmati et al., 2012; Bannon et al., 2006; Penades et al. 2005; Roh et al., 2005; Tukel et al., 2012; Okasha et al., 2000; Aigner et al., 2007; Bucci et al., 2007). However, the current study findings were inconsistent with the results by some other studies (Abbruzzese, Ferri, & Scarone, 1995; Hwang et al., 2007; Kitis et al., 2007; Nakao et al., 2009; Roth, Baribeau, Milovan, & O’Connor, 2004; Simpson et al., 2006; Ghadiri et al., 2006; Nedeljkovic et al., 2009; Cha et al., 2008).

Nedeljkovic et al. (2009) indicated that OCD washing did not differ significantly from healthy controls in planning, problem solving, and set-shifting. The researchers stated that the prefrontal cortex and especially the DLPFC were sensitive to WCST, which determines the set-shifting ability index (Nakano et al., 2008). Penades et al. (2005) in agreement with the current study results reported that despite controlling the depression effect, the patients with OCD were impaired in set-shifting, inhibition, and immediate non-verbal memory. The patients with OCD showed more preservative errors than the healthy ones (Roh et al., 2005). In the current study, the number of categories completed by the OCD washers was significantly lower, indicating weaker concept formation. This finding was also reported in some previous studies (Rao, Reddy, Kumar, Kandavel, and Chandrashekar, 2008).

Cognitive flexibility is the individual ability to shift from one task to another or change behavior when receiving negative feedback. To explain the results of the current study, it can be said that the observed inflexibility in the OCD washers can be a good reason for their severe repetitive, habitual, and compulsive behaviors.

Despite the negative feedback they receive, OCD washers are not able to easily change their repetitive behaviors. Since the set-shifting is dependent on understanding the abstractive rules, it is difficult for the patients with OCD to build an assumption and rightly examine it, probably because of the doubt they usually have. It is reasonable to explain persistent and inflexible thoughts and behaviors of the patients with OCD through executive function concept. Weaker cognitive flexibility and abstractive ability in the patients with OCD washing cause them to be more vulnerable to attention/distractibility. In WCST, lower number of completed categories indicates a worse abstractive ability; besides, lower conceptual level response and higher perseverative response scores show a cognitive inflexibility and more mental rigidity.

Results of the current research indicated that cognitive inhibition was significantly lower in the case group compared with the control group. In Stroop trials, OCD washers had a greater error rate, which means their failure rate to respond to incongruent trials was higher; also the patients’ increased interference effect indicated that they had to slow down significantly more when inhibiting incongruent information.

These findings were in accordance with the results of studies by Penades et al. (2007), Penades et al. (2005), Bannon et al. (2006), Bannon et al. (2002), Bohne, Keuthen, Tuschen-Caffier, and Wilhelm (2005), and Abramovitch et al. (2011); on the other hand, they were not in accordance with those of Moritz et al, (2002), GhamariGivi, Shaieghi, & Ghasemnejad, (2009), Nakao et al. (2005), and Rao et al. (2008). Patients with OCD had slower reaction time and made more errors in the interference condition of the Stroop task (Penades et al., 2007) compared with the control group. The mentioned incongruence in the findings is probably rooted in different factors including age, disorder severity, duration of the symptoms, medication effects, heterogeneity of the OCD symptoms, and the assessment tools. Finally, it can be suggested that, due to the impairment in the cognitive inhibition function, these individuals had a weak performance in suppressing the interference between congruent and incongruent responses. Cognitive inhibition in the patients with OCD caused controversy in the scientific community.

Another important finding of the current research was the difference in global performance of the groups in Go/No-Go Task, which indicated that the patients with OCD had a weaker behavioral inhibition compared with the control group. Bannon et al. (2002), showed the inhibition deficit in the patients with OCD using the Go/No-Go Task. Chamberlain et al. (2006) reported the motor response inhibition deficit in the patients with OCD. However, Ghamari Givi et al. (2009) showed no significant difference between the patients with OCD and the normal individuals using the stop-signal task.

The current study showed a significant increase in the number of omission errors in the OCD group compared with healthy individuals, which was in accordance with the previous studies results (Da Rocha et al., 2011), although some studies reported no significant difference in omission errors between the groups (Krishna et al,. 2011; Penades et al,. 2007; Watkins et al., 2005). The current study findings suggested that patients with OCD were impaired in sustained attention. In the current study, the patients with OCD committed significantly more commission errors than the healthy ones, indicating that individuals with OCD had a tendency toward inappropriate motor responses to non-target stimuli.

The increased number of commission errors in the current study was in accordance with some previous studies (Abramovitch et al., 2011; da Rocha et al., 2011; Ghisi et al., 2013; Penades et al., 2007); however, some studies reported no significant difference in the number of commission errors between the groups (Bohne, Savage, Deckersbach, Keuthen, & Wilhelm, 2008; Thomas, Gonsalvez, & Johnstone, 2014). Finally, the current study findings suggested the inability of the patients with OCD to properly inhibit obsessive thoughts, and environmental stimuli might be due to their cognitive and motor inhibition dysfunction. Mean reaction time in Go/No-Go Task was an indicator of processing speed. A review on previous studies showed a reduced processing speed in the patients with OCD (Abramovitch et al., 2011; Hashimoto et al., 2008; Penades et al., 2007). Similar results were obtained in the current study showing slower reaction time on Go/No-Go Task among the patients with OCD.

According to Lee and Kown (2003), there are two broad subtypes of obsessions: autogenous obsessions and reactive obsessions. The patients with OCD with autogenous obsessions (aggressive, religious, and sexual) showed a poorer performance in Go/No-Go Task than the patients with OCD with reactive obsessions (contamination, symmetry, and physical health) (Lee, Yost, & Telch, 2009). Van Boxtel, Van der Molen, Jennings, Cornelis and Brunia (2001), using the stop signal paradigm, indicated that the motor inhibition control is performed by the frontal cortex. Neuroimaging studies indicated the hyperactivity of the anterior cingulate cortex (ACC) during error commission of the no-go trials (Maltby, Tolin, & Worhunsky, 2005) and low frontal activity during no-go trials (Herrmann, Jacob, Unterecker & Fallgatter, 2003).

According to the research findings, the patients with OCD had weak performance in both cognitive and motor inhibitions, which was in accordance with the clinical experiences. Therefore, the repetitive nature of OCD symptoms may be the reason for difficulties in suppressing inappropriate actions. The most important characteristic of OCD washing is the disability to inhibit certain behaviors (e.g., washing hands or impulsive actions). This viewpoint can explain the OCD patients’ intense urge to perform compulsions, although they are aware of the meaninglessness of their behaviors (Robbins, Gillan, Smith, de Wit, Ersche, 2012).

The current study suggested that the patients with OCD were characterized regarding deficit in task control, which was also suggested by Kalanthroff, Anholt, Keren, and Henik (2013). Chamberlain et al. (2005) suggested that the cognitive inhibition deficit (ie, control over internal cognition) can lead to the obsessions, and the behavior inhibition deficit (ie, the control over external motor activity) can result in compulsions. However, generalization of the results to neuropsychological domain still demands more experimental and clinical studies.

The results of the current study on the digit span indicated that the patients with OCD were significantly impaired in working memory and global attention, compared to the healthy controls. The current study results were consistent with those of some previous studies (Sayin, Oral, Utku, Baysak, & Candansayar, 2010; Tukle et al., 2012); however, several studies did not find any significant differences between the 2 groups in digit span (Okasha et al., 2000; Moritz et al., 2002, Kuelz et al., 2004; Boldrini et al. 2005; Hashimoto et al., 2008; Segalàs et al., 2010). Notably, these differences can be attributed to the heterogeneity of OCD subtypes, severity of disorder, and the medication effect.

In addition, to the statistical significance, gaining the effect size could bring a deeper and more reliable understanding about the subjects. According to the definition of Cohen (1988) on effect size, the current study findings showed a large effect size in the neuropsychological functions. Effect size values, in accordance with significance level (P), were obtained for all the neuropsychological test variables, which indicated significant differences between the 2 groups. These results showed reduced performance in OCD washing. Previous meta-analytical studies reported different effect sizes of various executive function components (Shin et al., 2014; Abramovitch et al., 2013; Snyder et al., 2015). These differences in effect size can be probably explained through the heterogeneity of OCD subtypes and different methodological issues in previous studies.

Exploration for the correlation between the severity of OC symptoms and neuropsychological performance in OCD washing was the 2nd purpose of the current research. In the current study, the Y-BOCS scores did not show a significant correlation with any of the neuropsychological variables. The current study results were consistent with those of the previous studies (Bannon et al., 2006; Krishna et al., 2011; Roopesh et al., 2013). In contrast, results of several studies revealed the correlation between severity of OC symptoms and results of neuropsychological tests (Abramovitch et al., 2011; Nedeljkovic et al., 2009; Segalas et al., 2008).

No significant correlation between the severity of OC symptoms and neuropsychological functions indicated that the observed neuropsychological deficits were more trait-like factors rather than state-like factors (Bannon et al., 2006; Chamberlain et al., 2005; Roopesh et al., 2013). Therefore, the traits vs. state–like approaches to OCD are subject to controversy among many researchers. A few researchers indicated that response inhibition deficit was an endophenotype of OCD (Chamberlian et al., 2005; Morein-Zamir, Fineberg, Robbins & Sahakian, 2010). These results were in good agreement with the trait hypothesis stating that the executive functions had no dependence on symptoms. Hence, it can be expressed that set-shifting and response inhibition are the putative endophenotypes. The results of the current study were in accordance with neuroimaging data suggesting structure abnormalities and metabolism of prefrontal cortex in the patients with OCD.

One of the limitations of the current research was that the sample included only the adult patients with OCD washing; hence, the findings cannot be generalized to other age groups. Roth, Milovan, Baribeau, and O’Connor (2005) reported a difference in late-onset type and the early-onset type of OCD regarding neuropsychological functions. Therefore, it is suggested that the future investigations attempt to compare neuropsychological executive functions in different age groups, especially the children and adolescents. The sample size in both groups was relatively small.

It is suggested to use larger sample sizes to investigate and compare neuropsychological functions in future researches. It is also recommended that the future studies investigate and compare the neuropsychological deficits among patients with OCD washing with or without depression. Inclusion of other variables such as verbal and non-verbal memory and visuospatial abilities in the future researches is also recommended; therefore, they can be compared with other subtypes of the OCD. And also due to the inequality between male and female ratio, the gender differences were not investigated; hence, the gender-dependent variable can be focused in future studies.

Overall, it can be said that regarding neuropsychological features, the OCD washer group had some characteristics different from those of the control group. These findings were congruent with the neuroimaging studies, which indicated the impairments fronto-striatal network of the patients with OCD. Prefrontal cortex abnormalities play an undeniable role in high neurocognitive dysfunctions among the patients with OCD. It was concluded that executive function impairment, which was a core feature in OC washers, was trait-like in nature. Therefore, these findings could be applied in prevention, early diagnosis, and treatment.
